# Increased Time in Range and Fewer Missed Bolus Injections After Introduction of a Smart Connected Insulin Pen

**DOI:** 10.1089/dia.2019.0411

**Published:** 2020-10-06

**Authors:** Peter Adolfsson, Niels Væver Hartvig, Anne Kaas, Jonas Bech Møller, Jarl Hellman

**Affiliations:** ^1^ Department of Pediatrics, The Hospital of Halland, Kungsbacka, Sweden.; ^2^ Institute of Clinical Sciences, Sahlgrenska Academy at University of Gothenburg, Gothenburg, Sweden.; ^3^ Data Science, Novo Nordisk A/S, Søborg, Denmark.; ^4^ Medical & Science, and Novo Nordisk A/S, Søborg, Denmark.; ^5^ Digital Health, Novo Nordisk A/S, Søborg, Denmark.; ^6^ Department of Medical Sciences, Uppsala University, Uppsala, Sweden.

**Keywords:** Connected insulin pen, Time in range, Adherence, Hypoglycemia, Glycemic control

## Abstract

***Background:*** This observational study investigated whether the connected NovoPen^®^ 6 could influence insulin regimen management and glycemic control in people with type 1 diabetes (T1D) using a basal-bolus insulin regimen and continuous glucose monitoring in a real-world setting.

***Methods:*** Participants from 12 Swedish diabetes clinics downloaded pen data at each visit (final cohort: *n* = 94). Outcomes included time in range (TIR; sensor glucose 3.9–10.0 mmol/L), time in hyperglycemia (>10 mmol/L), and hypoglycemia (L1: 3.0– <3.9 mmol/L; L2: <3.0 mmol/L). Missed bolus dose (MBD) injections were meals without bolus injection within −15 and +60 min from the start of a meal. Outcomes were compared between the baseline and follow-up periods (≥5 health care professional visits). Data were analyzed from the first 14 days following each visit. For the TIR and total insulin dose analyses (*n* = 94), a linear mixed model was used, and for the MBD analysis (*n* = 81), a mixed Poisson model was used.

***Results:*** TIR significantly increased (+1.9 [0.8; 3.0]_95% CI_ h/day; *P* < 0.001) from baseline to follow-up period, with a corresponding reduction in time in hyperglycemia (−1.8 [−3.0; −0.6]_95% CI_ h/day; *P* = 0.003) and L2 hypoglycemia (−0.3 [−0.6; −0.1]_95% CI_ h/day; *P* = 0.005), and no change in time in L1 hypoglycemia. MBD injections decreased by 43% over the study (*P* = 0.002). Change in MBD injections corresponded to a decrease from 25% to 14% based on the assumption that participants had three main meals per day.

***Conclusions:*** Our study highlights the potential benefit on glycemic control and dosing behavior when reliable insulin dose data from a connected pen contribute to insulin management in people with T1D.

## Introduction

Globally, regardless of diabetes type, many people treated with insulin struggle to take their medication on time and also maintain their treatment regimen over extended periods of time.^1–4^ Difficulties with insulin dosing and inaccurate dose timing have been shown to result in poor glycemic control for people with diabetes.^5,6^ The impact of missed insulin injections on HbA_1c_ levels is well established,^7–12^ leading to an increasing risk of diabetes-related complications.^13,14^

Technological advances offer opportunities to optimize insulin delivery, reduce dosing errors, and improve regimen management. Diabetes treatment and care is moving toward accurate, real-time, high-quality data that are easily available to people with diabetes and health care professionals (HCPs). In the era of smart phones, connected continuous glucose monitoring (CGM) systems, and activity trackers, advancements in insulin pen device design is part of the future. Connected pens have the added ability to record insulin dose data, thereby improving convenience. The use of connected insulin pens alongside CGM may have the potential to facilitate and improve diabetes management.

Two types of CGM systems are available for diabetes self-management: real-time CGM (rtCGM) and intermittently scanned CGM (isCGM; also referred to as flash glucose monitoring). CGM, including isCGM (hereafter, unless stated, CGM includes both rtCGM and isCGM), is being used by an increasing number of people with type 1 diabetes (T1D).^15,16^

The key benefits of CGM include real-time data monitoring for people with T1D and access for them and their HCPs to complete glucose datasets^17^; both of which help people to achieve their target time spent within the acceptable range (time in range [TIR]: 3.9–10.0 mmol/L [70–180 mg/dL]) and avoid hyper- and hypoglycemia.^18^ Reductions in HbA_1c_ have been reported with CGM use in people on multiple daily injection (MDI) therapy,^19^ and continuous subcutaneous insulin infusion therapy,^20^ with reduced time spent in hypoglycemia,^20^ and improved hypoglycemia awareness.^21^

The connected insulin pen, NovoPen^®^ 6, administers insulin in 1 U dose increments, with a maximum dose of up to 60 U. The number of units of the last administered insulin dose and the time elapsed since administration is shown on an electronic display. The NovoPen^®^ 6 has a 5-year battery life, and collects and stores data on the date and time of injections and the number of units administered; these can be downloaded, using near field connectivity, to a centralized database on a computer-based data visualization program such as diasend^®^ (Glooko, Inc., CA). These data allow both users and HCPs to access and visualize insulin injection patterns over time, together with the information from home blood glucose meters and/or CGMs.

The connected NovoPen^®^ 6 has the potential to move dialogues regarding diabetes management with HCPs away from guessing about administered doses, toward true knowledge about missed doses, optimal injection time, and optimal dose size in relation to meals when combined with CGM. This could create a more complete picture of the current glycemic control, treatment, and disease state for people with T1D and HCPs. Therefore, the aim of this observational study was to investigate whether the connected NovoPen^®^ 6 could influence insulin regimen management and glycemic control in people with T1D using CGM in a real-world setting.

## Methods

### Study design and participants

This study was a one-arm, prospective, observational, proof-of-concept study, including 12 diabetes clinics in Sweden from May 2017 until April 2019. For the analyses presented here, study data from May 2017 to October 2018 only were used. Participants were continuously enrolled into the study from initiation until the complete data set was downloaded from the Glooko database. Swedish Ethics Committee approval (2019-01270) was obtained before any study-related activities. Written informed consent was obtained from each participant by Glooko, allowing Glooko to collect the participant's data and share it with Novo Nordisk for scientific purposes.

**FIG. 1.**
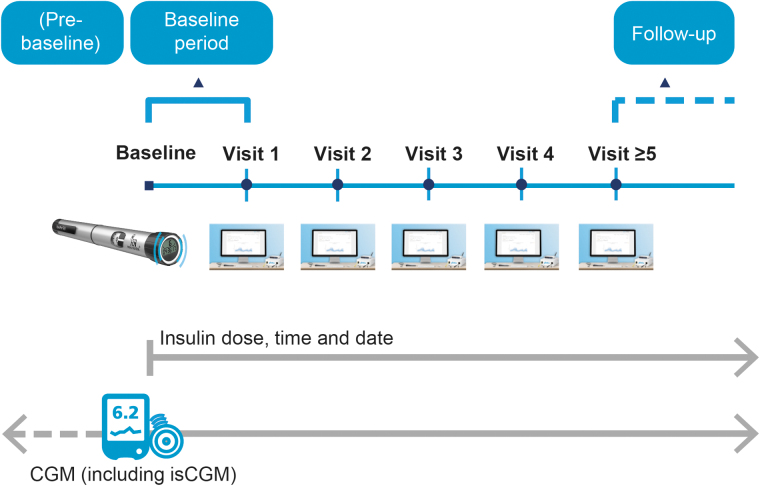
Study design. Prebaseline was the period before study commencement where participants were already using CGM, but without concurrent use of the NovoPen^®^ 6. CGM, continuous glucose monitoring; isCGM, intermittently scanned continuous glucose monitoring.

People with T1D using CGM, from the participating diabetes clinics, were included in the study at the discretion of their treating physician. At baseline, participants received a NovoPen^®^ 6 for basal and/or bolus insulin injections. Participants were blinded to the injection data overview during the baseline period (baseline until visit 1; Fig. 1). However, participants could still see their CGM data and they could also see the dose of their last injection displayed on the pen.

**FIG. 2.**
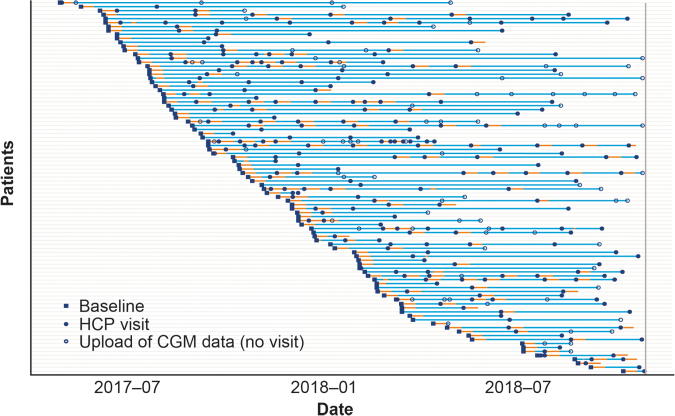
Upload of participant CGM data. Study period for each participant. A total of 94 participants are included in the TIR analysis. Blue lines indicate the period where data are available from the baseline date (blue square) to the last date with either CGM or insulin dosing data in the database. The filled blue circles indicate visits to the clinic, where data were downloaded and evaluated with the HCP. Orange lines indicate days with acceptable CGM data* within 1–14 days that are included in the primary analysis. The open blue circles are virtual uploads of CGM data that were not physical HCP visits. *CGM coverage of at least 70% per day. CGM, continuous glucose monitoring; HCP, health care professional; TIR, time in range.

At visit 1, the first set of injection data were downloaded at the clinic for discussion between the participant and HCP. As there is no difference in the operation of the NovoPen^®^ 6 compared with traditional durable pens, except for connectivity features, participants did not receive any structured education or training; individual centers may have provided informal training on the connectivity component of the pen at, or after, the first visit. Thereafter, follow-up visits were scheduled according to usual clinical practice; at each visit, pen and CGM data were downloaded, discussed and acted upon by the participant and HCP. CGM data were also uploaded between visits (Figs. 1 and 2).

Pen data could only be downloaded at the HCP's office (i.e., not at a participant's home). Data were downloaded from the Glooko database with anonymized participant IDs; data were then cleaned to ensure consistency and to avoid duplication (as detailed in the Results section).

### Outcomes

Glycemic summary measures and number of missed bolus dose (MBD) injections were compared between the blinded baseline period and a follow-up period. The follow-up period was defined as any point after the fifth HCP visit. Visit 5 was chosen as the earliest point for follow-up, as participants would on average have been in the study for ≥180 days, allowing for sufficient interaction with HCPs and discussion of available pen data. Days from baseline to visit 5 (Q1, median, and Q3) were 167, 196, and 260.

**FIG. 3.**
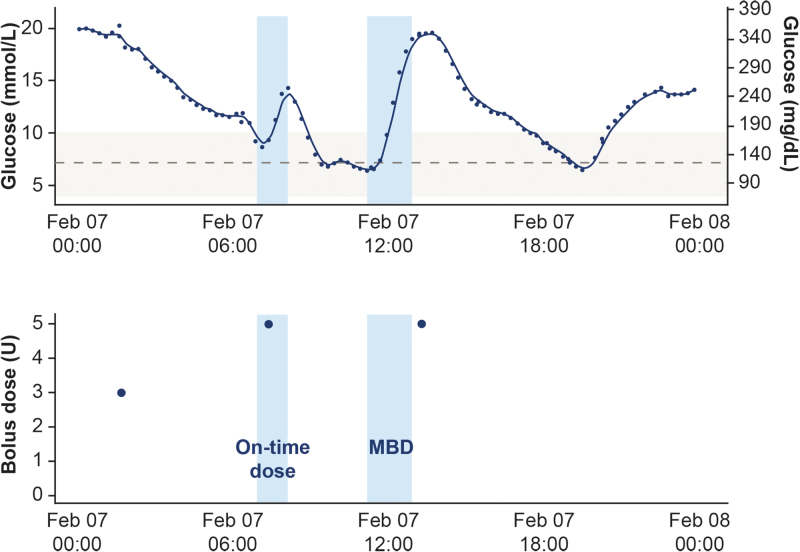
Detection of missed bolus insulin doses by the GRID algorithm. Example of a day with two meals detected. The solid dark blue line represents the CGM signal and the light blue shaded areas each represent a detected meal. The gray, dashed line represents a glucose level of 7.2 mmol/L (130 mg/dL) and the gray shaded area represents a target glycemic range of 3.9–10.0 mmol/L (70–180 mg/dL). Meals are detected when the CGM signal is ≥7.2 mmol/L (≥130 mg/dL) and with rate-of-change being sufficiently high over the last two to three readings corresponding. The blue circles in the lower figure indicate bolus doses. A bolus dose within 15 min before to 60 min after a meal starts is considered “on-time,” whereas a dose outside of this time window is considered a MBD. Male patient, aged 30 at baseline. CGM, continuous glucose monitoring; GRID, Glucose Rate Increase Detector; MBD, missed bolus dose.

TIR was defined as the time spent with sensor glucose within the acceptable range (3.9–10.0 mmol/L [70–180 mg/dL]). Additional outcomes included time spent in hyperglycemia (>10.0 mmol/L [>180 mg/dL]) and time spent in hypoglycemia, split into Level 1 (L1: 3.0– <3.9 mmol/L [54– <70 mg/dL]) and Level 2 (L2: <3.0 mmol/L [<54 mg/dL]). In addition, the total daily insulin (basal/bolus) dose, mean glucose level, and the coefficient of variation were measured.^22^ MBD was defined as meals with no bolus injection within a time window of 15 min before to 60 min after the start of a meal, as detected from the CGM signal by the clinically validated Glucose Rate Increase Detector (GRID) algorithm (Fig. 3).^23^ An “on-time” dose was defined as when a bolus insulin injection was detected within 15 min before and 60 min after the start of a meal.

Meals were detected when the CGM signal was ≥7.2 mmol/L (≥130 mg/dL) and the rate-of-change was ≥5.3 mmol/(L·h) [≥95 mg/(dL·h)] for the last two consecutive readings, or ≥5.0 mmol/(L·h) [≥90 mg/(dL·hour)] for two of the last three readings. The majority of participants used isCGM, which registered readings every 15 min. Therefore, the rtCGM signals, which registered readings every 5 min, were resampled to 15 min intervals. CGM profiles typically did not display all three meals per day, possibly due to low-carbohydrate or well-dosed meals that were indistinct in the CGM signal to the GRID algorithm. This was due to the algorithm being designed to detect meals that contained at least 40 g of carbohydrate.

### Statistical analyses

Data from the first 14 days following each clinic visit were used in the analyses in line with the international consensus on the use of CGM.^22^ Days with unacceptable CGM coverage (<70%) were excluded. Approximately, 2500 days' worth of CGM data with acceptable coverage were included.

Due to the variation in study duration and visit frequency observed between participants (Fig. 2), an initial analysis investigating whether there was a relationship between baseline participant characteristics and the variations in study duration and visit frequency was conducted.

TIR values were calculated both in hours, based on the time interval between readings, and as a percentage of all readings on a given day. Slight differences in the results from these two methods were expected due to sections with missing data or additional isCGM readings obtained when manually scanning the sensor.

For the TIR and total insulin dose analysis (*n* = 94), each day was aggregated to a single value for each response. A linear mixed model was applied with visit number (baseline, 1, 2, 3, 4, 5+) as fixed effect and participant and visit nested within participant as random effects. An exponential covariance function was used to model the correlation between days within a 14-day period. All 94 participants with acceptable CGM data were included in the analysis, and a total of 14 participants contributed with data from visit 5 or later (Supplementary Fig. S1).

All data from all participants were included in the analysis, as the linear mixed model allowed for unbalanced and missing data. Robustness with respect to the choice of follow-up period was evaluated by estimating the change from baseline for each single visit. The estimated difference between baseline and the follow-up period was obtained with 95% confidence intervals. The dosing data were analyzed on a logarithmic scale and estimates and confidence intervals converted to relative differences in percent.

For the MBD injection analysis, this was restricted to participants with bolus pen data (*n* = 81). The change in the mean number of MBD injections from baseline to the follow-up period (after five HCP visits) was analyzed using a generalized linear mixed model based on the Poisson distribution with visit number (baseline, 1, 2, 3, 4, 5+) as fixed effect, and participant and visit nested in participant as random effects, and using a logarithmic link-function. As for the TIR analysis, all 81 participants were included in the analysis. The number of participants achieving ≥5 visits was 10. Robustness with respect to the choice of follow-up period was evaluated by estimating the change from baseline for every visit.

A significance level of *P* < 0.05 was predefined for all statistical comparisons. All statistical models were verified based on residual plots (not shown).

## Results

### Study population

A total of 270 participants' IDs were downloaded from the Glooko database. However, some of the dose records in the database were duplicates from the same pen registered under different participant IDs, indicating that some participants had registered with multiple IDs (i.e., multiple e-mail addresses). Participant IDs sharing the same pen had to be relinked and inconsistent IDs or those IDs with unusable data were excluded, resulting in 224 participants remaining in the cohort.

Participant IDs were excluded from the cohort if they did not have both CGM and pen data, if they were children <18 years of age or if they did not have CGM data within 14 days of a visit. Children and adolescents were excluded from the analyses; some children had additional functionality to upload data from home, which meant that a clear link between upload points and clinic visits could not be established. This resulted in a final cohort of 94 participants, representing 35% of participant IDs received, included in the main analyses.

Of these 94 participants, 48 were men and 46 were women, with a mean age of 40.1 years (range 18–83 years). Eleven participants used CGM with 5 min intervals between readings and 83 used isCGM with 15 min intervals between readings. A total of 64 participants used NovoPen^®^ 6 for bolus insulin only, 17 for both basal and bolus insulin, and 5 for basal insulin only (7 participants did not have connected pen data in the 14-day period following a visit and 1 participant used biphasic insulin aspart 30, which is neither bolus nor basal insulin). The majority of participants with a basal insulin pen used insulin degludec (*n* = 21), with one participant using insulin detemir. Of participants with a bolus insulin pen, a total of 79 used insulin aspart as the bolus insulin, with one participant using human insulin and one participant using faster-acting insulin aspart.

The average study duration (the number of days from baseline to the last day with any relevant data [CGM or pen] before the download date from Glooko) was 223 days (range 14–487 days), with a mean of 27 days' data included per participant from day 1 to 14 after a visit. Visit frequency and time between visits varied between participants, as visits were scheduled according to clinical practice (mean number of visits in the study: 2.6 visits [range 0–11 visits]; mean time between visits: 71 days [range 1–319 days]). There was no indication of a relationship between baseline parameters (age, glycemic control, or insulin dose) and study duration or visit frequency.

### Glycemic summary measures and insulin dosing

**FIG. 4.**
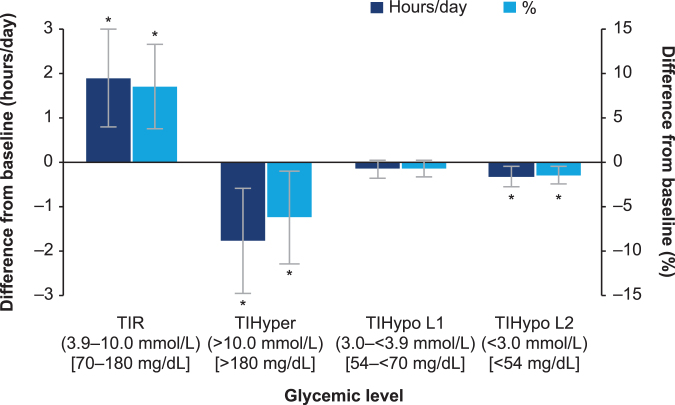
Mean difference in the time spent in glycemic ranges from baseline to after five HCP visits. *P < 0.05. Estimated mean difference in time spent in glycemic ranges with 95% CI. The difference is observed between baseline and ≥5 HCP visits. Baseline is the period after treatment initiation but before the first visit. Analysis is based on CGM data from a 14-day interval after each visit (≥70% coverage). Patients ≥18 years (*n* = 94) are included. CGM, continuous glucose monitoring; CI, confidence interval; HCP, health care professional; *n*, number; TIHyper, time in hyperglycemia; TIHypo L1, time in L1 hypoglycemia; TIHypo L2, time in L2 hypoglycemia; TIR, time in range.

Of the final cohort of 94 participants, 14 made ≥5 visits to a HCP (Supplementary Fig. S1). The number of participants with data at visits 1, 2, 3, and 4 was 57, 30, 21, and 15, respectively. The change in TIR was relatively stable from visit 4 onward, as shown in Supplementary Figure S1. A significant absolute increase in mean TIR of 1.9 [0.8; 3.0]_95% CI_ hours per day (*P* < 0.001) or 8.5% (percent of day) [3.7; 13.3]_95% CI_ (*P* < 0.001) was reported, from baseline to after five HCP visits (Table 1 and Fig. 4). This corresponded to a relative increase of about 21% of the mean baseline level. Accordingly, there was a significant reduction in mean time spent in hyperglycemia (>10.0 mmol/L [>180 mg/dL]) of −1.8 [−3.0; −0.6]_95% CI_ hours per day (*P* = 0.003) or −6.2% (percent of day) [−11.5; −1.0]_95% CI_ (*P* = 0.021) (Table 1 and Fig. 4).

**Table 1.** Baseline Level and Estimated Change at or After Five Health Care Professional Visits: Key Glycemic Summary Statistics

	Baseline level [95% CI]	Estimated change [95% CI]	P
TIR (3.9–10.0 mmol/L [70–180 mg/dL])	9.19 h [8.28; 10.10]	1.89 h [0.79; 2.99]	<0.001
	41.4% [37.4; 45.3]	8.5% [3.7; 13.3]	<0.001
TIHyper (>10.0 mmol/L [>180 mg/dL])	11.80 h [10.81; 12.79]	−1.78 h [−2.96; −0.60]	0.003
	53.5% [49.0; 58.1]	−6.2% [−11.5; −1.0]	0.021
TIHypo L1 (3.0– <3.9 mmol/L [54– <70 mg/dL])	0.69 h [0.55; 0.83]	−0.15 h [−0.36; 0.07]	0.181
	3.1% [2.5; 3.7]	−0.7% [−1.6; 0.2]	0.141
TIHypo L2 (<3.0 mmol/L [<54 mg/dL])	0.47 h [0.32; 0.61]	−0.33 h [−0.56; −0.10]	0.005
	2.1% [1.4; 2.7]	−1.5% [−2.5; −0.5]	0.004
Mean glucose	11.09 mmol/L [10.53; 11.65]	−0.34 mmol/L [−0.96; 0.28]	0.279
% Coefficient of variation	35.89% [34.33; 37.45]	−3.84% [−6.12; −1.56]	0.001

Estimated baseline level and change between visits ≥5 and baseline with 95% CI. Linear mixed model of TIR per day, with visit number (baseline, 1, 2, 3, 4, 5+) as fixed effect, participant and visit nested in participant as random effects, and with exponential covariance function. *N* = 94, visits = 231, CGM days = 2552. TIR values were calculated both in hours, based on the time interval between readings, and as a percentage of all readings on a given day. Slight differences in the results from these two methods may occur due; sections with missing data or additional CGM readings at the time of sensor scanning.

CGM, continuous glucose monitoring; CI, confidence interval; *N*, number; TIHyper, time in hyperglycemia; TIHypo L1, time in L1 hypoglycemia; TIHypo L2, time in L2 hypoglycemia; TIR, time in range.

There was no significant change in mean time spent in L1 hypoglycemia (3.0– <3.9 mmol/L [54– <70 mg/dL]; *P* = 0.181; Table 1 and Fig. 4). A significant reduction in L2 hypoglycemia (<3.0 mmol/L [<54 mg/dL]) of −0.3 [−0.6; −0.1]_95% CI_ hours per day (*P* = 0.005) or −1.5% (percent of day) [−2.5; −0.5]_95% CI_ (*P* = 0.004) was also observed (Table 1 and Fig. 4). While the mean glucose level did not change significantly (−0.34 mmol/L [−0.96; 0.28]_95% CI_), the coefficient of variation was significantly reduced by 3.8% [−6.1; −1.6]_95% CI_ from a baseline level of 35.9% (*P* = 0.001; Table 1).

In terms of bolus insulin dose (*n* = 81), there was a significant increase from baseline (25.1 U/day [22.0; 28.7]_95% CI_) to after five HCP visits of 28% [9.4; 49.5]_95% CI_ (*P* = 0.002). There was no significant change from baseline (24.2 U/day [19.8; 29.8]_95% CI_) in mean basal insulin dose (*n* = 22; change 11% [−7.2; 32.8]_95% CI_, *P* = 0.238).

### MBD injections

**FIG. 5.**
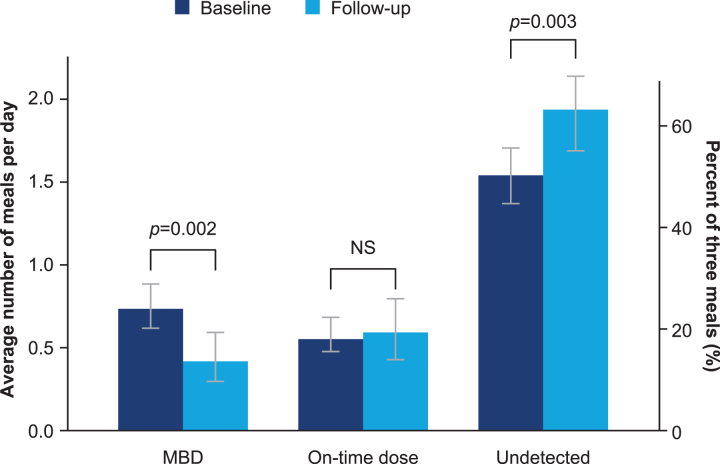
Mean number of daily meals and dosing behaviors from baseline to after 5 HCP visits. Estimated mean number of daily meals with 95% CI. MBD are meals with missed bolus doses. “On-time” doses are meals where a bolus dose is taken. Undetected are meals that are not detected by the CGM signal, assuming an average of three meals per day. CGM, continuous glucose monitoring; CI, confidence interval; HCP, health care professional; MBD, missed bolus dose; NS, not significant.

Eighty-one adults with T1D were included in the MBD analyses, which included 2747 detected meals; 10 participants in this analysis had ≥5 visits. A significant decrease of 43% (estimated relative change: −43.1% [−60.5; −18.0]_95% CI_, *P* = 0.002; Table 2) in the average daily number of MBD injections was observed from baseline (0.74 [0.62; 0.88]_95% CI_) to the follow-up period (0.42 [0.30; 0.60]_95% CI_, *P* = 0.002; Table 2 and Fig. 5). The change in MBD injections by visit is included in Supplementary Figure S2, which shows that a significant effect was seen from visit 3, and that it was relatively consistent from this point onward. Based on the assumption that participants have three main meals per day, the change in daily number of MBD injections corresponded to a decrease from 24.7% [20.8; 29.4]_95% CI_ (five meals with a missed dose per week) to 14.0% [9.9; 19.9]_95% CI_ (three meals with a missed dose per week) in MBD injections.

**Table 2.** Mean Number of Daily Meals and Dosing Behaviors from Baseline to After Five Health Care Professional Visits

	Baseline level [95% CI]		Visit ≥5 level [95% CI]		Estimated relative change [95% CI]	P
	Daily meals (*n*)	Proportion of 3 meals	Daily meals (*n*)	Proportion of 3 meals		
MBD	0.74 [0.62; 0.88]	24.7% [20.8; 29.4]	0.42 [0.30; 0.60]	14.1% [9.9; 19.9]	−43.1% [−60.5; −18.0]	0.002
“On-time” dose	0.57 [0.48; 0.69]	19.1% [15.9; 23.0]	0.59 [0.43; 0.80]	19.6% [14.5; 26.7]	2.7% [−24.7; 40.2]	0.865
Undetected meals^a^	1.54 [1.37; 1.70]	51.5% [45.6; 56.7]	1.94 [1.69; 2.14]	64.6% [56.4; 71.2]	25.4% [8.7; 43.5]	0.003

Estimated mean data and 95% CI based on a mixed Poisson model, with visit number (baseline, 1, 2, 3, 4, 5+) as fixed effect and participant and visit nested in participant as random effects.

^a^ Assuming three meals per day on average.

CI, confidence interval; HCP, health care professional; MBD, missed bolus dose; N, number.

## Discussion

Many people treated with insulin fail to reach their glycemic targets, which is known to increase the risk of microvascular and macrovascular complications, resulting in substantial morbidity and mortality.^13,14,24^ Our findings in this observational study in adults with T1D using CGM highlight the potential benefits on glycemic control and injection behavior when connected pen data contribute to insulin treatment. Previous studies have identified that an engaging and open dialogue between HCPs and people with T1D is highly important for optimal disease management, as it promotes collaboration, communication, and people's participation in their own treatment decisions.^25,26^ Our results suggest that the use of a connected pen might help to facilitate more informed dialogues between HCPs and people with T1D.

Over the course of the study, participants experienced less glucose variability, more TIR and less time in hyperglycemia and L2 hypoglycemia (<3.0 mmol/L [<54 mg/dL]); these changes occurred early, after five visits with a HCP. The recent Advanced Technologies & Treatments for Diabetes Congress consensus recommendations suggest that an approximate 5% increase in TIR is associated with clinically significant benefits.^27^ Two analyses found that an increase in TIR of 10% corresponded to a decrease in HbA_1c_ of ∼0.5%–0.8% (5–9 mmol/mol).^28,29^ Therefore, even small improvements in TIR can yield corresponding improvements in HbA_1c_, and result in clinically significant benefits. Based on these analyses, the 8.5% increase in TIR reported here would be expected to correspond to an improvement in HbA_1c_ of ∼0.4–0.7% (4–7 mmol/mol).

Notably, in the current study, time in hypoglycemia did not increase, while time in L2 hypoglycemia (<3.0 mmol/L [<54 mg/dL]) decreased. Mean glucose levels showed only a slight, but nonsignificant, decrease (HbA_1c_ data were not recorded). However, glucose variability decreased significantly, indicating that the improved TIR was primarily due to more consistent and stable glucose levels (CGM profiles) rather than a general reduction across the day. The significant increase in the injected bolus dose from baseline to the follow-up period (28%) may have contributed to the improved TIR and stable profiles.

The finding of only a small, nonsignificant, increase in basal dose may be due to relatively few participants in the study using connected pens for their basal insulin. A previous study has demonstrated that improvements in glycemic control and glycemic variability were associated with an increased frequency of blood glucose testing and administration of bolus insulin.^30^ Other studies have demonstrated that fluctuations in blood glucose levels may contribute more to outcomes than constant high blood glucose concentrations.^31–35^ As measurements of HbA_1c_ do not reflect glycemic variability and target HbA_1c_ values may be achieved while still experiencing marked daily glycemic fluctuations, people with T1D may regard reducing glucose variability and improving TIR to be as important as achieving their target HbA_1c_ value.^36,37^

The study data also confirmed that missing bolus injections can be common for people with T1D, where on average at least 25% of meals had a missed dose, assuming three meals per day. This amounts to five meals with a missed dose per week on average. Supporting people to engage with and optimize their insulin regimens is a key challenge for HCPs, with educational interventions having limited effects.^38^ Studies have found significant correlations between HbA_1c_ levels and the number of missing bolus injections.^7,39^ A recent study observed that the rate of late or missed bolus injections was 27% in people using MDI therapy, and that missed bolus injections correlated with higher HbA_1c_ levels.^40^ In a study of youths receiving insulin pump therapy, two missed bolus injections each week was associated with an increase in HbA_1c_ of 0.5% (5 mmol/mol).^39^

Our findings indicate that a connected pen may help people to reduce the number of missed bolus injections and to properly adjust doses, thus leading to better glycemic control. Participants in our study achieved a greater number of well-dosed meals, with 43% fewer meals with missed doses through connected pen use. This suggests that evaluating past dosing data together with HCPs may have helped participants to remember and administer their meal-time doses, or to improve the timing of the dose relative to the meal. Taken together, our TIR and MBD data support the hypothesis that connected pens may support people with the management of their insulin treatment regimens.

Observational studies have inherent limitations, which should be considered when interpreting our results. As this was a short single-arm study, we cannot conclude with certainty that the use of a connected pen directly improved glycemic control, and other factors may have played a role. We are confident that the effect seen was not due to the use of CGM alone; an analysis of a subset of participants with confirmed CGM data in the database, at least 3 weeks before baseline (i.e., existing CGM users), demonstrated virtually the same results (data not shown). Longer studies are warranted to adequately assess effect durability.

Since visits were conducted according to local clinical practice, the time in study, number of HCP visits, and time between visits varied considerably between participants. This may have resulted in a selection bias; however, data analyses did not find any evidence of relationships between baseline participant characteristics and study time or visit frequency. In addition, the visit frequency for most participants seemed to be higher than what would be expected in normal clinical practice.^41^ Participants may have been more engaged in their glucose profiles and insulin dose patterns and therefore visited their HCPs more frequently to download and discuss their glucose and insulin dosing data. Equally, this may be explained by the initial need for more frequent visits when introducing a new technology. Further investigation of the effect of at-home data download capacity on visit frequency might permit digital consultations and reduce the need for clinic visits.

There was limited access to background participant information within the dataset, such as diabetes status and comorbidities. Many participant IDs (65%) were excluded from the analyses during data cleaning. Furthermore, as data were unbalanced, the use of a linear mixed model with different levels of correlations was necessary.^42^ This allowed all data to be used, and by including participant as a random effect, it ensured that each participant had their own baseline level, and the estimated change from baseline to visit 5 can be interpreted as the mean change from this level. For the TIR analysis, only 14 participants had data from visit 5 onward (Supplementary Fig. S1), meaning that the estimated change is based on the data from these, while the remaining data are used to estimate the random variation between participants, visits, and days and thus contribute to degrees of freedom in the variance estimates.

This is the first real-world insight into outcomes with connected pen use in clinical practice. Data presented here were subject to acknowledged limitations, and larger, controlled follow-up studies are needed. Nonetheless, our findings suggest that connected pens such as the NovoPen^®^ 6 have the potential to improve glycemic control, decrease glucose variability and increase treatment concordance in people with T1D, addressing the large unmet need for optimal insulin treatment.

## Supplementary Material

Supplemental data
