# Supplementary material for: Increased Time in Range and Fewer Missed Bolus Injections After Introduction of a Smart Connected Insulin Pen
Source: Diabetes Technol Ther. 2020 Oct 6;22(10):709–18. doi: 10.1089/dia.2019.0411 (PMC7591375; doi:10.1089/dia.2019.0411)
Supplement: Supplemental data [file Supp_FigS1-S2.pdf]

Supplementary Data

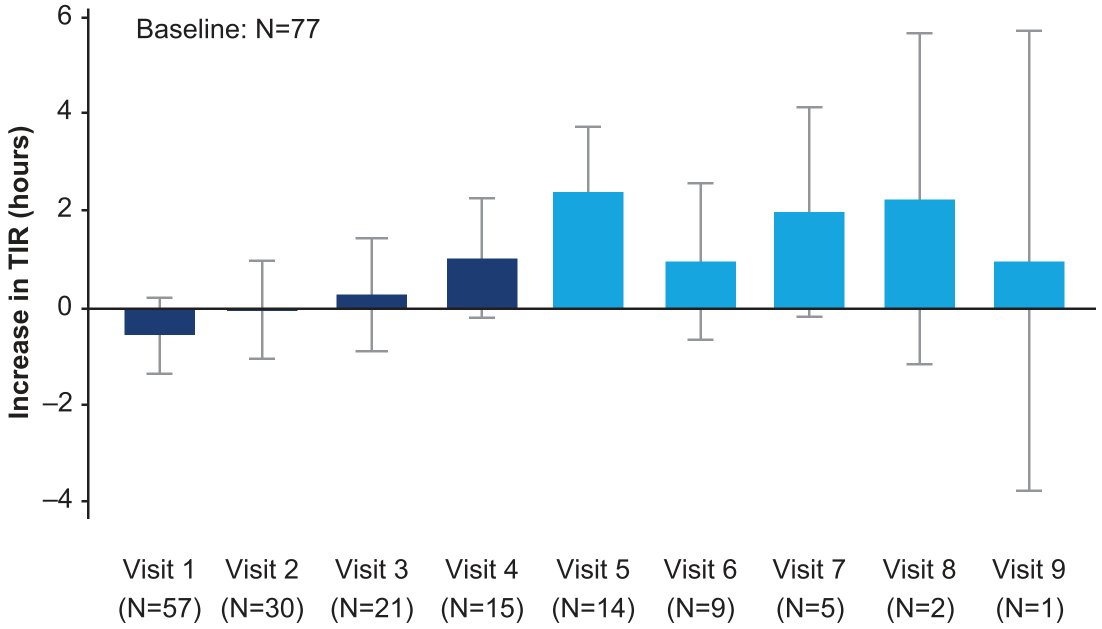

SUPPLEMENTARY FIG. S1. Estimated mean difference in time spent range (3.9–10.0 mmol/L [70–180 mg/dL]) with 95% CI. The difference is observed between baseline and visit period. Baseline is the period after treatment initiation, but before the first visit. Visits were conducted according to clinical practice. The analysis is based on CGM data from a 14-day interval after each visit (≥70% coverage per day). The dark blue bars represent visits 1–4. The light blue bars represent visits 5–9. CGM, continuous glucose monitoring; CI, confidence interval; *n*, number; TIR, time in range.

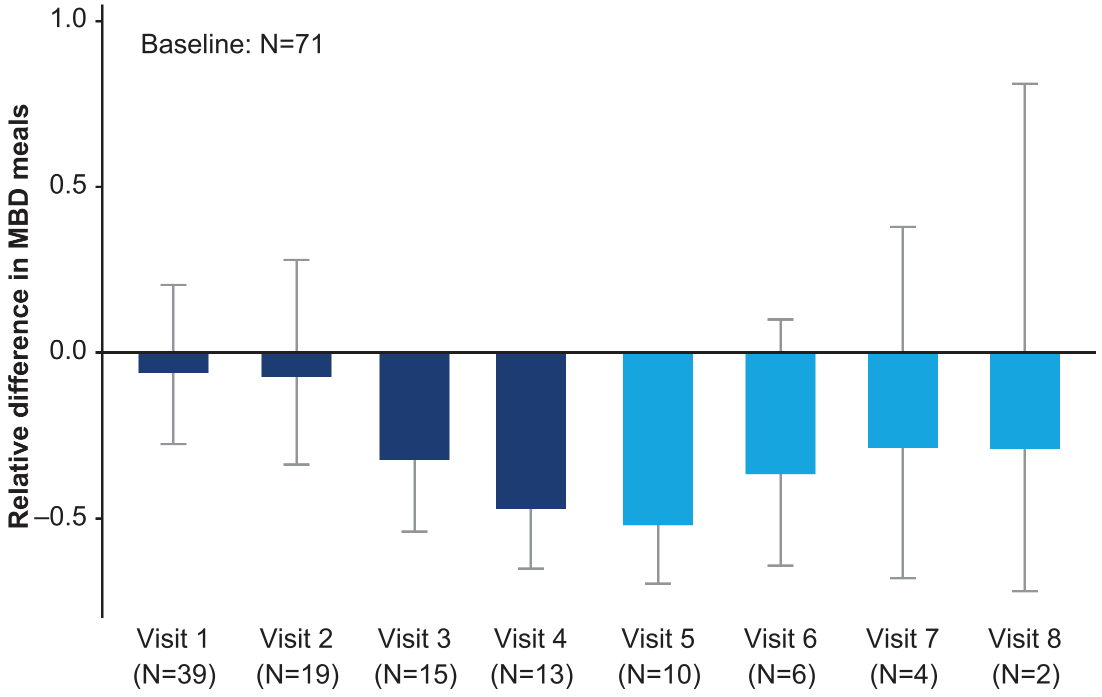

SUPPLEMENTARY FIG. S2. Estimated mean difference of daily MBD meals with 95% CI. The dark blue bars represent visits 1– 5. The light blue bars represent visits 5–8. MBD, missed bolus dose.
